# Role of Intestinal Microbiota on Gut Homeostasis and Rheumatoid Arthritis

**DOI:** 10.1155/2021/8167283

**Published:** 2021-06-04

**Authors:** Mingxin Li, Fang Wang

**Affiliations:** ^1^Department of Traumatic Orthopaedics, Tianjin Hospital, 406 Jiefangnan Road 406, Tianjin 300210, China; ^2^College of Mechanical Engineering, Tianjin University of Science and Technology, The Key Laboratory of Integrated Design and On-Line Monitoring of Light Industrial and Food Engineering Machinery and Equipment in Tianjin, Tianjin 300222, China

## Abstract

Rheumatoid arthritis (RA) is a chronic inflammatory disease that is immune mediated. Patients typically present with synovial inflammation, which gradually deteriorates to investigate severe cartilage and bone damage, affecting an individual's ability to perform basic tasks and impairing the quality of life. When evaluated against healthy controls, patients with RA have notable variations within the constituents of the gut microbiota. The human gastrointestinal tract mucosa is colonized by trillions of commensal microbacteria, which are key actors in the initiation, upkeep, and operation of the host immune system. Gut microbiota dysbiosis can adversely influence the immune system both locally and throughout the host, thus predisposing the host to a number of pathologies, including RA. Proximal intestinal immunomodulatory cells, situated in specific locales within the intestine, are a promising intermediary through which the gastrointestinal microbiota can influence the pathogenesis and progression of RA. In the early stages of the disease, the microbiota appear to differ from those present in healthy controls. This difference may reflect potential autoimmune mechanisms. Research studies evaluating intestinal microbiota have demonstrated that RA is associated with a bacterial population growth or with a decline when judged against control groups. The aim of this review is to examine the studies that connect intestinal dysbiosis with the autoimmune pathways implicated in the pathogenesis of RA.

## 1. Introduction

Rheumatoid arthritis (RA) is a chronic, immune-mediated disease with an inflammatory pathology. Clinical features include joint swelling, joint tenderness, synovial joint damage, and manifestations of systemic inflammation. RA typically leads to marked disability and an early demise [1, 2]. Premature death results predominantly from excess cardiovascular phenomena that occur autonomously from conventional cardiovascular risk factors and that are linked with augmented systemic inflammation [3]. The exact cause of RA is incompletely elucidated. The contemporary perspective is that, in individuals who have a genetic predisposition to RA, environmental agents provoke a pathological triggering of the immune system that ultimately leads to the clinical syndrome [3].

The European League Against Rheumatism has suggested certain nomenclature for the distinct preclinical stages of RA progression, which do not automatically fall in series and are not incongruous [4]. The risk of RA is heightened by the interplay between the genetic and environmental elements, together with the existence of autoantibodies.

In terms of environmental contributors, the gut microbiota has been shown in mice to participate in the development of arthritis [4, 5]. Increasing numbers of publications have recognized aberrations in gut microbiota constituents as major players in multiple pathological processes, the most notable being chronic inflammatory conditions [6, 7]. Recent work has indicated that some change in the constituents of the gut microbiota leads to a dysbiotic state that impacts the governance of immune function and fosters a proinflammatory phenotype [8]. Consequences include enhanced vulnerability to autoimmune disorders (e.g., inflammatory bowel disease, systemic inflammatory arthritis, and connective tissue pathologies), dysmetabolic syndromes, and malignancies [7].

It has recently been demonstrated in humans that immunoglobulin A (IgA) anticitrullinated protein antibodies (ACPAs) can be identified for many years before the clinical presentation of arthritis [9]. This finding implies that RA is derived from mucosal areas, such as the intestine and the mouth. The clinical efficacy of antibacterial agents (e.g., minocycline or salazosulfapyridine) in some patients with RA supports the theory that the gut and oral microbial populations are associated with RA [10].

The purpose of this review is to evaluate studies that have demonstrated changes in the constituents of gut microbiota in patients with RA. Furthermore, the presented evidence connects intestinal dysbiosis with the autoimmune pathways implicated in the pathogenesis of RA.

## 2. Immunopathogenesis of RA

Elucidation of the complicated molecular pathways that contribute to the development of RA remains fraught with difficulty. A range of environmental elements may silently trigger a pathological stimulation of the immune system in individuals who have a genetic predisposition [11]. This untoward immune system galvanization may lead to the synthesis of clinically silent autoantibodies, such as rheumatoid factor (RF) or ACPA. There may then be a period of minimal symptomatology or a preclinical stage, which generally heralds the clinically evident presentation of features that represent typical RA [12].

Earlier work has proposed that immune irregularities, such as immunomodulatory cell (IC) stimulation or suppression, that occur at regional and then systemic levels exist in individuals who are predisposed toward RA [13]. Populations of T and B lymphocyte subsets, in terms of both number and activity, are linked with the mechanisms underlying RA onset [3]. Potential antigens include type II collagen, proteoglycans, and cartilage protein gp39 [14, 15].

The joints of people with RA are complex structures in which intrinsic and adaptive immune cell populations, together with local cell types (i.e., synoviocytes and chondrocytes), participate in the disease process [16]. Autoantibodies and autoreactive T cells identified within the joints reflect the dysregulatory state of the immune system. The manufacture of autoreactive B cells is the most evident change in the immune system of an individual with RA, and this process develops well before the onset of clinical disease. Essential for the pathogenesis of RA, autoreactive B cells generate ACPAs and RFs. Within lymphoid regions, heightened T cell stimulation is associated with the perseverance of switched memory B cells [17]. Fc receptor-like protein 4 is a subset of memory B cells that expresses IgA. It is involved in the regional autoimmune response that promotes joint damage in individuals with RA via receptor stimulation of nuclear factor-*κ*B ligand expression [18].

Many immune system aberrations in RA develop at the level of the mucosa. During RA onset, the immune response within the intestinal mucosa is notably amplified, antigen-presenting cells (APCs) display aberrant stimulation, and immune tolerance is disrupted [19]. Diminished immunity in the mucosa to citrullinated proteins or peptides together with novel B cell conscription is an essential element of antirheumatic treatment responses in individuals at a premature stage of the disease [20].

Many cell types participate in the disease process of RA. Within the synovium, dendritic cells are located predominantly within lymphocytic clusters and the peripheral vasculature, implying that they are derived from the circulation. APCs express major histocompatibility complex alleles that associate extracellular peptides with CD4^+^ T cells, thus impelling the liberation of proinflammatory cytokines that trigger the manufacture of antibodies from B cells [21].

In rats with collagen-induced arthritis (CIA), CD8^+^ and CD4^+^ cell populations were elevated in Peyer's patches compared with the levels in control animals [22]. In mice with CIA, Sundstrom et al. [23] demonstrated that CD4^+^ T cells in the lamina propria were stimulated before the clinical development of arthritis. This change developed after notable upregulation of interleukin-17A (IL-17A), tumor necrosis factor-alpha (TNF-*α*), and granulocyte-macrophage colony-stimulating factor. The clinical gravity of the arthritis was notably diminished in the absence of Th17 [23]. Th17 differentiation is bolstered by an environment that contains macrophage-derived and dendritic cell-derived transforming growth factor-*β* and interleukin-1*β*, IL-6, IL-21, and IL-23. These factors also inhibit the differentiation of regulatory T cells, thus causing T cell homeostasis to incline toward the inflammatory process [24].

Posttranslational modifications (PTMs) are vital for protein function and antigenicity. Citrullination involves the modification of arginine into citrulline, a reaction catalyzed by peptidyl arginine deiminases; this reaction forms the principal PTM linked with self-antigen identification in RA [21]. Citrulline may change protein configuration and produce novel epitopes linked with the manufacture of ACPAs. Repertoire sequencing before clinical presentation in individuals with RA has demonstrated that this immune phenomenon commences in a highly limited fashion and then magnifies during a time period of several months to possibly years. Epitope dissemination, from the first identified epitope, expands to trigger reactivity to a range of epitopes before RA is diagnosed [25, 26].

The distribution of epitopes trends toward more citrullinated moieties, which is in keeping with the theory that an individual antigen, although not necessarily the identical antigen, initiates the immune response [12]. The levels of ACPAs increase, and the variation in epitopes increases before clinical presentation. ACPAs may encompass the isotypes IgG, IgA, and IgM, and a changed glycosylation condition bestows an augmented binding affinity for the Fc receptor and citrullinated antigens [27]. ACPAs can then act as pathogens per se by stimulating either macrophages or osteoclasts, a reaction mediated by the manufacture of immune complexes and Fc receptor association or, potentially, through an attachment to citrullinated vimentin in the cell membrane, which exacerbates bone loss [27].

Blood analysis of individuals with early RA who have been prescribed antirheumatic treatment has demonstrated that the titers of ACPA, IgA, and IgM swiftly decline, a change associated with reduced pathological activity [11]. The first recognition of antibodies in a subcohort of patients with RA was in the form of citrullinated antigens, but studies now suggest that citrullinated epitopes of numerous autoantigens and antigens originating from microorganisms can be identified by antibodies that are extremely specific for RA [11].

Genetic elements alone are clearly not solely responsible for RA [28], and the contribution of additional risk factors requires deeper investigation. It has been postulated that, during the preclinical stage of RA, an interplay among microbes, other candidate environmental elements (e.g., diet, physical or emotional pressures), and host factors arises at the mucosa, precipitating mucosal inflammation and the disruption of immune tolerance. Mucosal inflammation may then itself promote local and ultimately systemic immune dysregulation. Involved mechanisms may encompass molecular imitation or may enable immediate autoimmunity to the host's own antigens [29, 30].

Additional studies have indicated that the gut microbiota may represent a substantial experimental element in the pathogenesis of RA [31]. Changes in certain mucosal territories imply that microbial factors may influence the local mucosal immune response, thus also contributing to the early phase of RA development [32].

Variations in the microbiota constituent populations and their frequency (i.e., dysbiosis) can stimulate a number of autoimmune and inflammatory pathologies through a loss of equilibrium in the T cell subpopulation (e.g., Th1, Th2, Th17, and Treg cells) [16]. At the mucosal level, citrullinated microbial antigens and molecular mimicry, Toll-like receptor (TLR) signals, and additional intrinsic immune stimulators and hazard signals may be present [32]. Microbiological organisms linked with the mucosa influence members of the immune system (i.e., neutrophils, dendritic cells, and macrophages), viewing them as pathogenic and inflicting injury. This response leads to inflammation, the liberation of cytokines, an increase in chemokine levels, and autoantigen synthesis. Thus, potential engagement occurs among the gut microbiota, ICs, and the pathogenesis and progression of RA—a situation that has gained much attention recently.

## 3. General Introduction to Intestinal Microbiota

The most sizeable population of colonized bacteria within the human body is situated in the gastrointestinal tract. Each individual contains several hundred species [33], of which more than 90% are members of the *Bacteroidetes* and *Firmicutes* phyla. However, additional phyla (i.e., *Proteobacteria*, *Actinobacteria*, *Fusobacteria*, *Verrucomicrobia*, and *Cyanobacteria*) are also integral players in the upkeep of governance of microbiota homeostasis [30]. The microbiota perform many activities, but a key contribution is that of maintaining host immune system homeostasis. As a consequence, homeostasis may be influenced by any change in the microbiota population [34].

The adult configuration of the microbiota is formed immediately after birth [31]. Within the bacterial population, a number of advantageous symbiotic organisms contribute to homeostasis in a benign and mutually beneficial fashion. Also present are sensitive bacteria that are adversely impacted in the presence of pathologies, pathogenic members of the population that may induce disease, and therapeutic organisms that can assist in restoring the status quo after change [31].

The population of bacteria in the gut can be affected by diet, probiotics, prebiotics, antibiotics, exogenous enzymes, fecal microbiota transplantation (FMT), and additional environmental elements [35]. The microbiota work in concert with the intestinal interface to perform essential activities relating to the safeguarding of the immune homeostasis. Two key elements that may fluctuate and affect barrier robustness and its operation, with consequences on the governance of intestinal permeability, are diet and intestinal microbiota. These elements may permit external antigens to traverse the interface from the gut cavity into the host [33, 36]. These factors are both a consequence of lifestyle, which implies that environmental elements can affect the integrity of the intestinal interface's operations and therefore affect the immune response and the development of conditions such as RA [33].

The gut microbiota have been implicated in the pathogenesis of numerous intestinal and extraintestinal disorders [37]. Furthermore, dysbiosis of the microorganisms within the gut is tightly linked to the intestinal mucosal immune system, which has been associated with autoimmune conditions, including RA. Indeed, changes within the types (i.e., taxa, phyla, or genera) and prevalence of organisms exist; variations within this bacterial population develop at a premature stage of the disease [38], although the pathways underlying the pathogenesis of the changes remain unclear at both the cellular and molecular levels.

## 4. Evidence from Animal Models

Several rodent models of arthritis, including models of SKG, IL-1ra^−/−^, K/BxN, and CIA, have demonstrated the significance of the intestinal microbiota in the pathogenesis of RA [39, 40]. If mice are raised in a germ-free environment or administered antimicrobials, arthritis does not develop [39]. In germ-free-reared mice, the injection of certain microorganisms can precipitate arthritis [40], implying that the gut microbiota are instrumental in disease development. Specifically, some studies have demonstrated an exacerbation of RA in microbiota-colonized SKG mice [39, 41]. Another report in germ-free SKG mice monocolonized with *Prevotella copri* showed that arthritis could be triggered by a fungal injection [39]. Despite some research reporting an association of *Prevotella* species with the pathogenesis of arthritis, other publications have postulated that *Prevotella* is actually an advantageous, rather than a disease-inducing, species [42, 43].

One study compared bacterial types and proposed that immunological features cannot be determined by the phylum to which the bacterium belongs; this study emphasized the need to delineate traits at the level of the individual species [44]. Marietta et al. [44] assessed two species of *Prevotella* in relation to arthritis prophylaxis and therapy in HLA-DA8 mice. The capacity to influence the immune response was inconstant between the two strains. *Prevotella histicola* repressed the onset of arthritis by influencing the immune response (i.e., the governance of dendritic cells and the production of Treg cells), leading to suppression of the Th17 response and a decrease in inflammatory cytokines (i.e., IL-2, IL-17, and TNF). Conversely, *Prevotella melaninogenica* induced no alterations in cytokine titers and failed to inhibit the onset of arthritis. *P*. *histicola* was evaluated in a DBA/1 murine model, which showed that animals receiving this strain acquired less severe arthritis than the animals in the control cohort. These results clarify the fact that an individual *Prevotella* strain can behave in either a positive or an adverse fashion, according to the setting. This variation may be a reason that *Prevotella* species are plentiful in healthy microbiota, and it implies that only some strains have disease-inducing traits.

Segmented filamentous bacteria, which are gut commensals, are able to provoke robust gastrointestinal and systemic follicular helper T (TFH) cell responses, which lead to a surge in autoantibody manufacturing in K/BxN mice [45]. In SKG mice that have received curdlan, an IL-23-dependent decrease in intestinal goblet cells leads to impaired epithelial barrier operation. Furthermore, naïve SKG mice display fecal dysbiosis that also relies on IL-23. IL-23a is intrinsically expressed within the small intestine of naïve SKG animals but is lacking in the gut of naïve BALB/c or germ-free SKG mice [40]. In SKG mice, IL-23 prefers the outgrowth of spondyloarthritis-linked pathobionts, such as *Bacteroidaceae*, *Porphyromonadaceae*, and *Prevotellaceae*, and diminishes the reinforcement for homeostatic-generating microbiota, including *Clostridiaceae* and *Lachnospiraceae* [46]. In mice, the host mucosal-microbial boundary likely contributes substantially to IL-23-dependent mechanisms in the development of arthritis.

IL-1 receptor antagonist knockout (IL-1rn^−/−^) mice spontaneously acquire T cell-mediated arthritis under certain pathogen-free circumstances, forming another experimental murine model [47]. These animals fail to present with arthritis in a germ-free habitat. If the mice are monocolonized with *Lactobacillus bifidus*, arthritis is precipitated.

A study by Rogier et al. [48] demonstrated that the changed microbiota in RA is typified by a large prevalence of *Helicobacter* species and a modest presence of *Ruminococcus* species. Therapy with tobramycin diminished the quantity of commensal microorganisms, including *Helicobacter* species, and inhibited the development of arthritis in IL-1ra -/-^−/−^ rodents. In addition, when IL-1ra and TLR4 double-knockout mice were examined, dysbiosis in IL-1ra^−/−^ mice was demonstrated to be TLR4 dependent in nature [48].

A T cell receptor transgenic murine model of inflammatory arthritis, the K/BxN scenario, offers straightforward discernment between the initiation and effector phases [49]. K/BxN T cell receptor transgenic mice present with inflammatory joint disease associated with elevated autoantibodies toward glucose-6-phosphate isomerase [50]. When established in a germ-free setting, the mice fail to develop arthritis; diminished Th17 cell populations can be observed within the small intestine and spleen [51]. The addition of a monocolony of segmented filamentous organisms is enough to generate Th17 cell-dependent arthritis in these mice.

CIA models are frequently used to explore the mechanisms underlying RA. In this setting, alterations in the gut microbiota develop in the early, immune-priming phase, before clinical presentation of disease [4]. In one study, *Firmicutes* and *Proteobacteria* phyla formed the key actors within the intestinal microbiota; the prevalence of *Bacteroides* was diminished in mice with RA compared with levels in naïve DBA/1 mice [52].

Additional work has explored the microbiome of DBA/1 mice in those developing resistance or already resistant to CIA after immunization with type II collagen. Compared with the controls, mice with arthritis had a notably reduced spectrum of bacterial types; this reduction did not occur in the mice showing resistance to arthritis. The mice vulnerable to CIA had the greatest diversity of phyla before the clinical presentation of disease; the most prevalent phyla were *Firmicutes*, *Bacteroidetes*, and *Proteobacteria*. *Lactobacillaceae* were also more prevalent in the animals susceptible to CIA; the genus *Lactobacillus* was markedly more plentiful after the generation of arthritis in the CIA-susceptible versus the CIA-resistant animals [53]. Jubair et al. [54] also documented that CIA can be stimulated by intestinal dysbiosis through mucosal immune responses. In that study, dysbiosis and inflammatory mucosal reactions developed before CIA onset [54]. Jubair et al. [54] also decreased the microbiota using broad-spectrum antimicrobials in a time-dependent fashion. This reduction in microbiota had a maximal effect on the gravity of arthritis when implemented late after the booster injection, whereas microbiota depletion before the immunization had a more modest influence on the disease. The two antimicrobial prescriptions both resulted in the late synthesis of anti-type II collagen antibodies. However, only late administration decreased the glycosylation and complement-fixing capacity of anti-type II collagen antibodies [54].

The results of this study reinforce the conclusion that the host mucosal-microbial boundary is a major participant in enhancing inflammation in concert with the actions of the microbiota, thus triggering autoantibody glycosylation and provoking antigen-antibody-mediated joint disease. Therapy with antimicrobials mitigated disease gravity and diminished anti-type II collagen antibody and serum inflammatory cytokine titers. Thus, some commensal microbiota from the gastrointestinal tract are adequate to precipitate arthritis in murine models, although more detailed evaluations are required to pinpoint effects that may be implicated in arthritis for specific organisms.

## 5. Dysbiosis and Intestinal Microbiota in Humans with RA

Studies from the final 30 years of the 20th century have indicated quantitative alterations in certain species of microorganisms, including *Clostridium perfringens*, *Bacteroides*, *Prevotella*, and *Porphyromonas*, in individuals with RA [55]. More recent research reinforces the theory that the microorganism population within the gastrointestinal tract is a key actor in the pathogenesis of joint disease in humans. The mechanisms underlying bacterial involvement are almost certainly numerous, and proposals have encompassed stimulation of APCs by influencing TLRs or NOD-like receptors (NLRs), enzymatic initiation of peptide citrullination, mimicry of antigens, changes in the permeability of the gut mucosa, governance of the host immune system (e.g., activating T cell differentiation), and augmentation of mucosal inflammation via Th17-mediated pathways. Many case-control studies have demonstrated that the constituents of the intestinal microbiota in individuals with RA are varied (Table 1).

Using 16S RNA/DNA sequencing techniques, some studies have shown relatively enhanced numbers of *P*. *copri* in individuals, compared with healthy controls [56, 67]. Curiously, this relative increase was seldom identified in patients with RA who were well into the course of the disease or receiving long-term treatment; it was also rare in patients with a psoriatic form of arthritis [67]. The relative plentitude of *P*. *copri* was inversely associated with the existence of common epitope risk alleles, implying that the contents of the human gut microbiota could, to some extent, rely on the host's genetic material and reflecting the presence of a dysbiosis before the manifestation of the clinical phenotype [67]. An additional study reported that Japanese individuals early in RA harbored an augmented quantity of *Prevotella*, particularly *P*. *copri*, together with a reduced prevalence of intestinal *Bacteroides* [39]. This study recognized that *P*. *copri* itself had a notable ability to stimulate the production of Th17 cell-related cytokines, specifically IL-6 and IL-23. Excess amounts of *Prevotella* species have also been linked to heightened mucosal inflammation, which is mediated by Th17 pathways. This finding is in keeping with the notable propensity for *Prevotella* species to steer Th17 immune responses *in vitro* [39].

Another study has documented the increased intestinal prevalence of *Prevotella* species, encompassing *P*. *copri*, in patients with preclinical RA in European nations, also implying that dysbiosis predates the onset of arthritis [47]. Transplantation of a human microbiota from patients with RA in whom *Prevotella* was overwhelmingly prevalent into an animal model of arthritis led to severe disease; this disease transition failed to occur after transplantation of the microbiota from healthy controls [39]. Thus, an epitope bestowing cross-reactivity to antigens associated with arthritis may be transferred by *P*. *copri*. Moreover, a prophylactic influence of the *Prevotella* species (e.g., *P*. *histicola*) has been demonstrated in murine models [44].

However, additional genera may contribute to the generation of inflammatory pathology. Vaahtovuo et al. [68] reviewed the microbiota contents from patients with untreated premature RA or fibromyalgia using flow cytometry, 16S rRNA hybridization, and DNA staining. In the patients with RA, the genera *Bifidobacterium* and *Eubacterium rectale*-*Clostridium coccoides* from the *Bacteroides fragilis* subgroup were decreased, which is consistent with earlier reports about individuals with Crohn's disease [70]. Work in China, founded on metagenomic shotgun sequencing, reported increased quantities of *Lactobacillus salivarius* in the intestine, on the teeth, and in the saliva of patients with RA [69]. However, *Haemophilus* species were decreased at all three locations. The prevalence of intestinal *P*. *copri* was greater in the initial year after clinical presentation, and notably, the dysbiosis detected in individuals with RA was incompletely resolved after therapy with disease-modifying agents [69]. Moreover, Liu et al. [61] documented that fecal *Lactobacillus* species were more plentiful in patients with RA in China compared with healthy controls [61]. Additional microorganisms (e.g., *Collinsella aerofaciens*) have exacerbated arthritis in murine models [57]. Chen et al. [57] noted that, in contrast to healthy controls, individuals with RA demonstrated reduced microbial variation within the gut, which was associated with autoantibody titers and length of disease [57]. Notably, methotrexate increased the number and variation of microbial species in patients with RA [57].

The main gut microbiota implicated in early RA and in its pathogenesis include *P*. *copri*, *L*. *salivarius*, and *Collinsella*. A relative increase of *Collinsella* was identified in patients with RA. One way in which *Collinsella* causes the disease is by enhancing gut permeability, as seen by decreased tight junction protein expression. The bacteria also affect the epithelial release of IL-17A [57].

In patients with de novo RA, the increased prevalence of *Prevotella* within the gastrointestinal tract has replaced *B*. *fragilis*, bacteria with notable Treg activity [67]. Increased counts of *P*. *copri* and analogous species are associated with poor titers of advantageous organisms, and this change may inhibit the immune system and the breakdown of vitamins into components absorbed into the circulation [71].

When exploring the effect of diet on RA, the influence of the intestinal bacteria should be taken into account [72]. *Bacteroides* species are linked with a diet containing substantial quantities of protein and animal fat; high-carbohydrate diets are related to the prevalence of *Prevotella* species [73]. Research has indicated that Mediterranean or vegan styles of eating diminish inflammation and enhance physical function and energy [74], although studies have not assessed parameters that quantify disease activity [75]. These studies also failed to establish whether the alteration in intestinal bacterial content ameliorated RA. The underlying rationale for the various potential arthritogenic organisms involves the host's genetic predisposition and environmental factors (e.g., diet). Additional studies are required to elucidate the role of intestinal dysbiosis and specifically changes in individual species in autoimmunity. Eventually, research must assess whether targeting intestinal microbiota abnormalities in those considered to be high risk for RA is successful in safeguarding against clinical disease presentation.

## 6. Future Directions

The results of studies evaluating the contribution of microbiota vary among the early disease course, disease before the use of immunosuppressive treatment, and mature disease with long-term treatment [69]. The 16S profiling technique identifies the relative plentitude of the range of species, thus providing a ratio, although the results may be vulnerable to prejudices from a number of intrinsic elements (e.g., bowel habits) [55]. A need exists for a more quantitative assessment of the microbiome and for detailed temporal metagenome-wide association research to investigate the operational ability of the microbiota within the host. This type of assessment will help resolve issues that require meticulous appraisal, including the directionality of dysbiosis. Additional microbiome studies should emphasize strain-level recognition of organisms and surmount the restrictions of analytical methods founded on 16S, which may lack accuracy [76].

To date, several dysbioses have been documented in the microbiota of patients with RA compared with healthy controls [39, 47, 67]. Microbiota from the intestine of individuals with RA has stimulated or worsened pathological phenotypes in RA [39]. Numerous questions must be addressed before a definite causal association between the human microbiome and the pathogenesis of RA can be confirmed. Comprehending these pathways is essential to improve the effectiveness of therapy and to guide patient-centric care. The flexibility of the biome may permit local or systemic maneuvering of specific gut microbiota linked with host pathologies [77]; this possibility engenders the conjecture that such interventions could alter treatment approaches in patients with RA. If the results from this area of research are valid, then transference into the clinical arena would generate de novo prophylactic or therapeutic initiatives.

## Figures and Tables

**Table 1:** Altered composition of the gut microbiota in humans with RA.

Author (year)	Increased bacteria	Decreased bacteria	Technology	Reference
Alpizar-Rodriguez et al. (2019)	*Prevotellaceae*, *Prevotella* spp.		16S rRNA sequencing	[56]
Chen et al. (2016)	*Collinsella aerofaciens* and *Eggerthella lenta*	*Faecalibacterium*	16S ribosomal DNA	[57]
Chiang et al. (2019)	*Verrucomicrobia*, *Akkermansia*		16S rRNA sequencing	[58]
Eerola et al. (1994)		Anaerobic bacteria: *Bacteroides*, *Prevotella*, and *Porphyromonas*	Gas-liquid chromatography of bacterial CFAs	[59]
Kishikawa et al. (2019)	*Prevotella*		Whole-genome shotgun	[60]
Liu et al. (2013)	*Lactobacillus*		Quantitative real-time PCR	[61]
Lee et al. (2019)	*Lactobacilli*, *Prevotella*	*Bacteroides*, *Bifidobacterium*, *Bacteroidetes*/*Firmicutes*	16S rRNA sequencing	[62]
Muñiz Pedrogo et al. (2019)	*Clostridiaceae*		Metagenomic shotgun sequencing	[63]
Maeda et al. (2016)	*Prevotella* (*Prevotella copri*)	*Bacteroides*	16S rRNA sequencing	[39]
Rodrigues et al. (2019)	*Bacteroides*, *Prevotella*	*Clostridium leptum*	qPCR	[64]
Shinebaum et al. (1987)	*Clostridium perfringens*, coliform		Estimation of bacterial counts in fecal culture	[65]
Sun et al. (2019)	*Bacteroides*, *Escherichia*-*Shigella*	*Lactobacillus*, *Alloprevotella*, *Enterobacter*, *Odoribacter*	16S rRNA sequencing	[66]
Scher et al. (2013)	*Prevotella* (*Prevotella copri*)	*Bacteroides*	16S rRNA sequencing	[67]
Vaahtovuo et al. (2008)		*Bacteroides*-*Porphyromonas*-*Prevotella*, *Bacteroides fragilis*, *Eubacterium rectale-Clostridium coccoides*	Flow cytometry,16S rRNA hybridization, and DNA staining	[68]
Zhang et al. (2015)	*Lactobacillus salivarius*	*Haemophilus* spp.	Metagenomic shotgun sequencing	[69]

## Data Availability

The data of this manuscript are available from the corresponding author upon reasonable request.
